# Is quinoa‐farming sustainable in marginal environments? Social, economical and environmental aspects

**DOI:** 10.1002/jsfa.70657

**Published:** 2026-04-27

**Authors:** Anna Tabea Mengen, Damir Esenaliev, Jakhongir Babadjanov, Lukas John, Sandra M. Schmöckel, Botir Khaitov

**Affiliations:** ^1^ Department Physiology of Yield Stability, Institute of Crop Science University of Hohenheim Stuttgart Germany; ^2^ The Leibniz Institute of Vegetable and Ornamental Crops (IGZ) and ISDC‐International Security and Development Center Großbeeren and Berlin Germany; ^3^ FAO Uzbekistan Tashkent Uzbekistan; ^4^ Cluster of Excellence, GreenRobust University of Hohenheim Stuttgart Germany; ^5^ Tashkent State Agrarian University Tashkent Uzbekistan

**Keywords:** agriculture, *Chenopodium quinoa*, economic and social considerations, environmental, marginal lands

## Abstract

Quinoa (*Chenopodium quinoa* Willd.) is an Andean grain crop introduced as a novel crop to many parts of the world in recent years. Recognized for nutritious seeds and high abiotic stress tolerance, it has been promoted as an element of climate‐resilient agriculture, particularly in marginal environments. Successfully introducing new crops to sustainably increase and diversify agricultural production in such settings depends on environmental, economic and social factors of the agricultural context. This review critically assesses the potential and challenges of introducing quinoa as an alternative crop, focusing on marginal lands in Central Asia. We highlight quinoas broad genetic base to abiotic stresses such as drought, salinity and temperature extremes and discuss how it can be leveraged by breeding for marginal environments. Environmental effects, economic aspects and initial experiences with quinoa cultivation in Central Asia are discussed. Furthermore, we discuss broader socioeconomic implications, including land tenure and migration in marginal regions. We conclude that quinoa holds potential for improving sustainable agriculture in marginal environments, when integrated policies and long‐term support enable its benefits for sustainable agricultural development. © 2026 The Author(s). *Journal of the Science of Food and Agriculture* published by John Wiley & Sons Ltd on behalf of Society of Chemical Industry.

## INTRODUCTION

Feeding the growing world population with sufficient and nutritious food is one of the most important challenges of our time. As a result of climate change, global crop yields are projected to decline beyond 2030, while arable land is shrinking and land degradation is on the rise.^1^ Agricultural production is affected by land degradation, increasing climate variability with extreme rainfall patterns and temperatures, which puts whole regions at risk of food insecurity.^2^ Therefore, the development of resilient agricultural systems that allow sustainable production in marginal environments is of utmost importance for supporting global food security.

Globally, marginal lands account for approximately 21% (2.74 billion hectares) of the total land area of 13.5 billion hectares.^3^ Of the 1.56 billion hectares currently used for agriculture, around 224 to 300 million hectares are considered agriculturally marginal. Demographically, about 1.75 billion people, or 38% of the global rural population, live in remote, less‐favoured, and marginal agricultural areas. Of these, approximately 1.6 billion reside in developing countries.

Marginal land refers to areas where farming is not cost‐effective because of challenging environmental conditions.^4^ These lands are typically found in rural regions where natural constraints make agriculture more difficult and expensive. Developing such economically or environmentally disadvantaged areas is important to preserve their social and economic value, support food and energy production, and maintain ecosystem services. Marginal land is usually identified using two main approaches: one based on economic indicators and the other on biophysical conditions such as soil, climate and terrain. Recommended uses for marginal land include high nature value farming, growing bioenergy crops and afforestation. Because the perception of marginal land can change over time and across regions, flexible policies and practical, nature‐based solutions are needed to ensure their sustainable and non‐degrading use. In this review, we focus on marginal land and environments in the sense of areas where biophysical constraints limit the profitability of food crops otherwise common to the area (Fig. 1).

**Figure 1:**
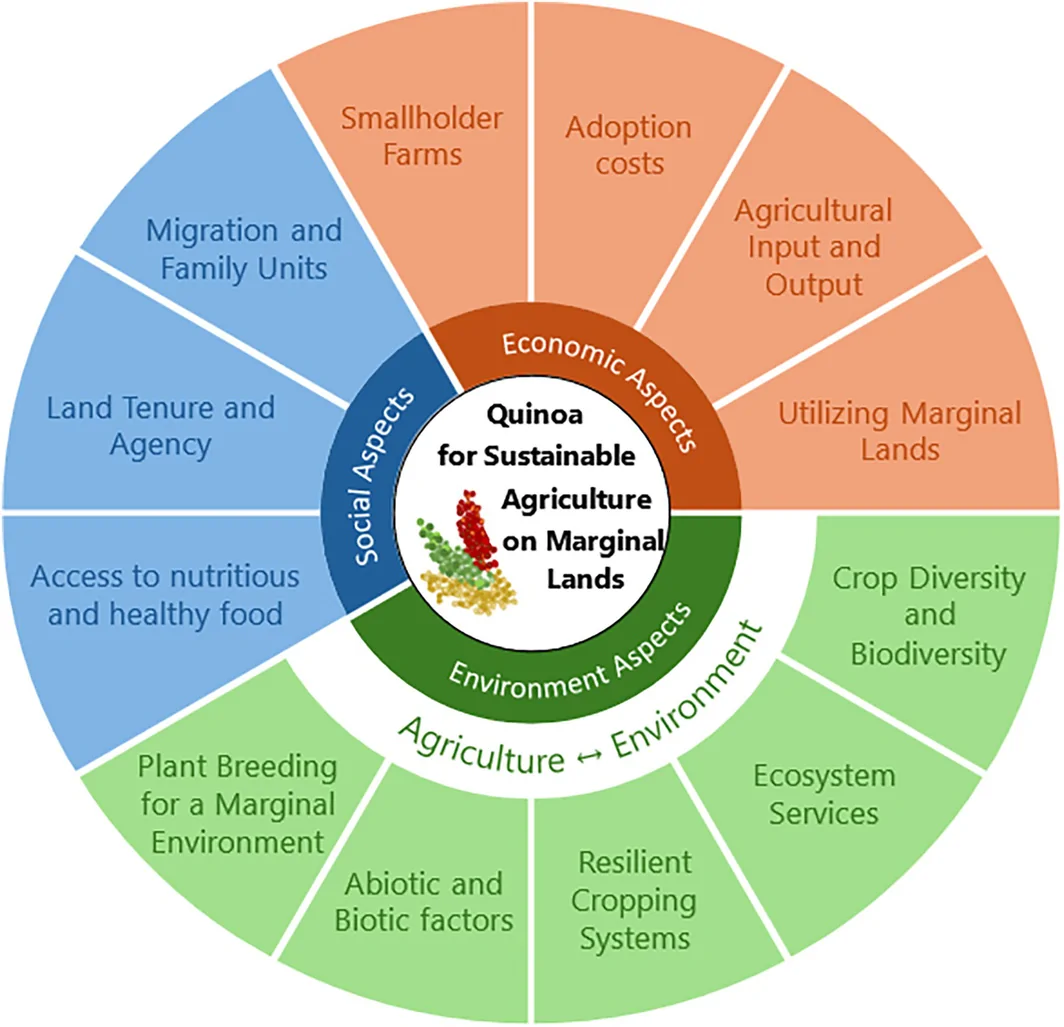
Quinoa may facilitate sustainable agriculture on marginal lands, particularly if economic, social and environmental aspects are considered.

Although sustainability as a term is used nearly inflationary, the concept of sustainability remains vague and its definition context dependent. Across disciplines, it has emerged to include three components: social, economical and environmental aspects.^5^ Applied in the context of agriculture, these components can be conceptualized as goals of respective categories with strategies and fields of actions, potentially inter‐ and counteracting, contributing towards these goals, which different stakeholders may prioritize differently.^6^ According to the Food and Agriculture Organization (FAO),^7^ sustainable agriculture has the goal to make nutritious food accessible for everyone in a way that meets both current and future generations' needs. It should be environmentally non‐degrading, economically viable and strive towards social equity.^7^ Sustainably intensifying agriculture by increasing yields and environmental performance on existing farmland is a challenge that needs to be addressed by diverse scientific disciplines, including not only plant and soil sciences, but also economic and social sciences. Taking more marginal lands into production and cultivation of underutilized species may hold potential contributing to sustainable intensification of agriculture.^8^

Quinoa (*Chenopodium quinoa* Willd.) is an annual seed crop originating in South America which was domesticated more than 5000 years ago in the Andes. It is one of the underutilized crops gaining more attention regarding its potentials for cultivation in challenging environments worldwide in recent years. The FAO declared 2013 as the year of Quinoa and while the main producing countries are still Bolivia and Peru, quinoa was the crop newly introduced to the largest number of countries between 2012 and 2019.^9^ Quinoa has been titled a ‘superfood’ for its nutritional qualities with high‐quality protein containing lysin and other essential amino acids.^10^ Because of its domestication history in different regions, quinoa is highly genetically diverse and developed different ecotypes adapted to various environments.^11^ Quinoa has been shown to be adaptable to marginal environments different from the Andean area under various adverse conditions.^12^, ^13^ Stable yields in such scenarios makes it a candidate for diversifying cropping systems for food security.^14^ In this light, Quinoa is discussed as having huge potential as a valuable crop for marginal environments worldwide, among others the arid, sandy and saline conditions of Central Asia.^15^

Central Asia represents unique agricultural systems with various challenges, such as salinity, desertification and drought. Climate change and its effects on subsistence farming are becoming increasingly critical, highlighting the urgent need for climate‐resilient crops and the adoption of climate‐smart agricultural technologies. Climate variability is anticipated to significantly threaten agricultural output and food accessibility, with projections indicating a decline in potential yield at the farm level. Intensified drought, desertification and salinization have already contributed to degrading millions of hectares of land in the region over the past decade.^16^ The region's arid climate, coupled with extensive irrigation practices, has led to widespread salinization of soils, significantly reducing agricultural productivity. One promising strategy for soil restoration in this increasingly salt‐affected region is the cultivation of halophytes that can rehabilitate degraded soils at the same time as contributing to food security.

This review explores the opportunities quinoa provides for sustainably increasing and diversifying crop production in marginal environments, discussing how quinoa can contribute to all three dimensions of sustainable agriculture. We therefore include environmental and agronomic aspects, as well as economic and social contexts, with a focus on Central Asia and the Aral Sea Basin as an example region to critically assess quinoa's potential in these agricultural contexts.

## ENVIRONMENTAL ASPECTS

The environmental aspects refer to those factors affecting quinoa‐based agriculture, such as abiotic factors (water deficit (drought), temperature extremes, degraded soils and specifically soil salinity) and biotic factors, and also include those being affected by quinoa‐based agriculture, such as ecosystem services. This section therefore also covers agronomic aspects, examples of quinoa cultivation in Central Asia and considerations for breeding quinoa for marginal environments.

### Water deficit

Water scarcity is a major constraint in marginal environments, limiting plant productivity and increasing production risks.^17^, ^18^ Quinoa is increasingly explored as a crop for water‐limited systems because of its drought resilience, efficient water use and highly nutritious grain. This resilience stems from a combination of morphological, physiological, biochemical and molecular traits. Deep root systems improve access to soil moisture,^19^ whereas careful stomatal control maintains photosynthesis under high vapour pressure deficits and low available water content.^20^ Osmotic adjustment through solute accumulation (e.g. K^+^, proline) sustains turgor under drying conditions.^21^ At the molecular level, drought induces antioxidant enzymes and expression of stress‐responsive genes involved in abscisic acid signalling and reactive oxygen species scavenging.^22^ Quinoa can also shift from anisohydric to more conservative water‐use under progressive stress, reducing transpiration at the same time as preserving CO_2_ uptake.^23^ It can maintain reproductive development and resume growth after rehydration, making it well‐suited for dryland and rainfed systems under climate pressure.^20^ Importantly, quinoa's broad genetic background underpins the intraspecific variation in these traits.^24^, ^25^, ^26^ Genotypes differ widely in their transpiration regulation, osmotic sensitivity, root architecture, gene expression dynamics and, most critically, in their ability to maintain yield under water deficit.^27^, ^28^ Therefore, identifying suitable genotypes for a target environment remains paramount.

### Temperature extremes

Besides drought, temperature extremes, both low and high, can be a feature of marginal environments and pose critical constraints to crop production. Quinoa shows considerable thermal adaptability, having been cultivated from sea level to over 4000 m above sea level across diverse climatic zones. Cold tolerance is largely genotype‐dependent: highland ecotypes are adapted to withstand sub‐zero night temperatures during early development, aided by mechanisms such as increased sugar accumulation, membrane stabilization and cold‐responsive gene expression.^29^, ^30^ However, thermal stress is not limited to early growth stages. Elevated temperatures, particularly during reproductive development, represent a major challenge to quinoa cultivation. Heat stress around the time of anthesis can severely reduce fertilization and seed set, with even short heatwaves causing substantial yield losses^31^, ^32^). The sensitivity during flowering is linked to pollen viability, anther dehiscence, stigma receptivity and the temporal regulation of reproductive processes.^33^ All of these can be disrupted by high temperature. Although quinoa is often described as moderately heat‐tolerant, its reproductive stages are especially vulnerable. Fuentes *et al*.^34^ speculated that quinoa in the Atacama Desert produce lower grain‐yield as a result of temperatures in the range 31–35°C during flowering. Nonetheless, physiological and biochemical heat tolerance adaptations such as downregulation of photosynthesis‐related genes and large shifts in lipids and flavonoids have been observed in some genotypes. This heat tolerance is associated with the regulation of heat stress transcription factors and purine metabolism, which together help initiate a rapid stress response.^35^ These traits, combined with genetic variation in flowering time, phenological plasticity and heat‐responsive gene expression, form the basis for improving tolerance. Breeding efforts have begun to identify heat‐resilient genetic relatives of quinoa (*Chenopodium berlandieri*) that can maintain yield under elevated temperatures, with the goal of creating tolerant, high‐yielding crosses.^36^ Yet, much of this diversity remains underexplored. In regions such as Central Asia, where temperature fluctuations and heatwaves increasingly intersect with key developmental windows, this presents both a challenge and an opportunity. Flowering time must be carefully managed through context‐specific sowing strategies to avoid exposure to peak heat. At the same time, targeted screening and selection of genotypes with stable reproductive performance under stress will be essential.

### Salinity and degraded soil

Quinoa is traditionally cultivated in the Peruvian and Bolivian Altiplano, characterized by an arid climate, frost, saline, sandy soil, low organic matter and nutrient contents, and erosion.^37^ Although quinoa responds positively to increased nitrogen levels through fertilization,^38^, ^39^ nitrogen‐supply in the Andes relied on llama manure, crop residues and a crop rotation for decades,^40^ confirming quinoa to be a valuable crop for production systems on poor soil conditions. Central Asian soils are generally characterized by low organic matter, excessive salinity and a neutral pH or slight alkalinity. Desertification, pollution, water and wind erosion are the main factors of deterioration of soil health in this region.^41^ Therefore, Central Asia presents a case where the introduction of quinoa into cropping systems may increase productivity on marginal lands.

Tackling soil salinity is crucial for the sustainable development of agriculture in Central Asia, requiring coordinated efforts in research, policy frameworks and practical field‐level interventions. As one of a few halophytic crops, quinoa is a great model crop for researching salinity tolerance in crops at the same time as developing diversifying options for cropping systems.^13^ The main salinity tolerance mechanisms of quinoa appear to be osmotic adjustment, osmoprotection, exclusion and xylem loading of Na^+^, and a reduced stomatal density.^42^ Sequestering Na^+^ in vacuoles is an energy‐efficient way of osmotic adjustment. To maintain the osmotic balance within a cell, the plant needs to keep a favourable Na^+^/K^+^ ratio and accumulate organic solutes. Quinoa was found to have a high uptake and retention of K^+^ and also found to maintain the Na^+^/K^+^ ratio in younger leaves lower than that in older leaves, where relatively more Na^+^ is accumulated.^43^ Regarding organic solutes, Olmos *et al*.^44^ suggested glycine‐betaine to have an important role. Osmoprotection relates to scavenging of reactive oxygen species, where organic solutes such as mannitol, proline and glycine‐betaine were shown to be increased under salinity. Furthermore, enzymatic activities, such as dehydrins might be involved in osmoprotection. Na^+^‐exclusion from the roots is seen as one of the most important salt tolerance mechanisms in plants, but studies suggested that Na^+^‐exclusion has a secondary role compared to quick loading of Na^+^ into the xylem for transport into the leaves.^42^ At a genetic level, underlying gene functions for these mechanisms remain unclear so far and few genes involved in salt tolerance have been cloned and characterized. Schmöckel *et al*.^45^ provided a list of 15 candidate genes for salt tolerance worthy of further investigation. Although quinoa in general is considered very salt tolerant and can keep yields in saline conditions, both the genotype and agronomic management affect its performance in such scenarios.^46^, ^47^, ^48^ Genetic variability for the trait has been shown and there is initial evidence that different ecotypes differ in their response to salt stress, regarding the timing and control of different mechanisms.^45^, ^49^ Consequently, the potential for cultivating quinoa in the salt affected areas of Central Asia is given, but the gap between the investigation of underlying molecular mechanisms and the application of results in practice has yet to be closed.

### Biotic stress

Although the focus is often on abiotic stresses in quinoa, biotic factors cannot be neglected when discussing quinoa cultivation. The most important disease in quinoa is downy mildew, caused by *Peronospora variabilis* Gäum., resulting in severe yield losses. It is not only endemic to South America, but also has been reported in various other locations.^50^ Management of the disease may include fungicides, although the more sustainable option would be resistance breeding because genetic variation in tolerance to the pathogen already exists.^51^ Furthermore, quinoa can be subject to a range of further fungal and bacterial diseases, as well as nematodes and few viruses. Proper characterization of the diseases is often lacking but becomes more important with globally increasing quinoa production.^52^ Regarding Arthropods, *Eurysacca quinoae* is the major pest in the original cultivation areas, but further insects, depending on the area, infest quinoa as well.^53^ With cultivation in new regions, pests related to weedy relatives of quinoa, such as *Chenopodium album*, also present as a risk.^54^ Worldwide, sustainable quinoa production will require effective pest monitoring and application of combined strategies, implementing integrated pest management.^55^

### Environmental impact

The use of quinoa as a crop in marginal environments likely has implications on an environmental level, as will be discussed here briefly. Quinoa's ability to thrive on nutrient‐poor soil and low water availability should limit problems related to over‐fertilization and nitrogen‐run‐off, and depletion of available irrigation water. During the ‘quinoa‐boom’ (2010–2014), quinoa production in Bolivia heavily increased and large areas were cleared from natural vegetation to accommodate quinoa fields with increasingly mechanized production. Many of these areas were soon abandoned, leaving barren land prone to erosion.^37^ Such scenarios of a traditional system turning unsustainable are to be avoided when introducing quinoa to new environments. Bonifacio *et al*.^56^ and Barrientos‐Pérez *et al*.^57^ suggest to plant native shrub species as live barriers to wind erosion and use native legumes as cover crops to regain sustainability of the production system. Applying this concept of regenerative agriculture when introducing quinoa to begin with, matching shrubs and cover crops to local conditions, may facilitate sustainability of quinoa production and help establish it especially in extremely harsh conditions, such as around the Aral Sea Basin. Furthermore, intercropping quinoa with legumes can improve seed protein content and yield of the total system.^58^, ^59^ Integrating quinoa into agroforestry systems is another strategy for arid regions.^60^, ^61^

One major feature of quinoa is the saponin on the seed. Saponins are triterpenoid secondary metabolites that have soapy characteristics (they foam in water) and taste bitter. Thus, they have to be removed before human consumption. Although the benefits for saponins on the seeds have not been studied well, they are assumed to be a bird deterrent and potentially help the plant with pests and diseases.^62^ This would provide an opportunity to breaking pest cycles, which are particularly problematic in monoculture systems. Saponin removal involves abrasion, requiring specialized machinery or washing off, which uses ample amounts of water and labour and is contradictory to farming in marginal environments. Thus, research on varieties without saponins (non‐bitter quinoa) has been receiving more attention. This would generate seeds without saponins, which then does not require extensive cleaning. However, non‐bitter varieties adapted to marginal environments still need to be identified or generated through breeding. A marker has been identified, which should facilitate breeding in the near future.^63^

### Ecosystem services

Ecosystem services, comprising the various benefits that humans derive from ecosystems such as clean water, food and climate regulation, are well known, particularly for agricultural systems that include legumes as they fix nitrogen and thus provide nitrogen‐enriched soils. Quinoa's drought and salt tolerance mean that the soil structure is less prone to erosion compared to areas without crops. Quinoa is reported to have allelopathic effects,^64^ which may be useful in weed suppression or growth with companion plants in an intercropping system. Quinoa has epidermal bladder cells, which are known to provide anti‐herbivory characteristics,^65^ and this may help break pest cycles. As quinoa becomes better characterized in various cropping systems and pedoclimatic environments, its contribution to ecosystem services will become better understood. Particularly, the subsidy and monetization of ecosystems services by governmental organization/policy would facilitate economic benefits of diversifying agriculture using quinoa.

### Pioneering quinoa cultivation in Central Asia

Quinoa was first introduced to Central Asia in 2015 through the International Center for Biosaline Agriculture's (ICBA) support as a new climate resilient and nutrient dense pseudo cereal to enhance the food and also nutritional security. Climate resilience associated with good adaptability to various agro‐ecological environments (soils, rainfall, temperature and altitude) and abiotic stresses (drought and salinity) may help incorporate quinoa into the marginal areas as an alternative crop.^66^, ^67^ Table 1 summarizes case studies on quinoa yield in field experiments in different Central Asian countries.

**Table 1:** Quinoa grain yield in field experiments in Central Asian countries

Source	Country/region	Genotypes	Year and experimental conditions	Reported yield range
Shitikova *et al*. (2022)^68^	Russia, Moscow region	Brightest Brilliant Rainbow, Red Faro, Cherry Vanilla, Titicaca, Regalona	2017–2020	1.01–3.81 t ha^−1^
			Non‐podzolic soil, overall climatic conditions	
Jorfi *et al*. (2022)^66^	Iran, Khuzestan, Ahwaz	Giza 1, Q26, Titicaca	2018–2019	1.04–5.72 t ha^−1^
			Varying phosphate and zinc fertilization	
Khaitov *et al*. (2021)^69^	Uzbekistan, Karakalpakstan, Chimbay	Q1, Q2, Q3, Q4, Q5	2015–2016	0.94–2.54 t ha^−1^
			Saline soil (6–12 dS m^−1^)	
Bazile *et al*. (2016)^14^	Tajikistan	Regalona, Puno, Titicaca	2014	Average for three genotypes: 2.13 t ha^−1^
			Respective overall soil and climatic conditions	
	Kyrgyzstan			Average for two test locations and three genotypes: > 1.24 t ha^−1^

Approximately 92% of Central Asia's 392.7 million hectares comprises pasture or unexploited land, with only about 8% designated as arable land. In countries such as Uzbekistan and Turkmenistan, a significant portion of arable land is irrigated due to arid climates. Among Central Asian countries, Uzbekistan is facing serious environmental challenges, salinized irrigated lands (EC 5–15 dS m^−1^) have risen from 38% in 1990 to approximately 58% in 2018.^70^ As a result of its strong adaptability to local agroclimatic conditions, quinoa holds potential as an alternative for rehabilitating salt‐affected farmlands that are no longer economically viable for cultivating traditional crops.^71^ These attributes highlight its strong potential for dryland agroecosystems, particularly with respect to addressing the growing challenges of climate change and food insecurity in the region.^72^

Quinoa cultivation in Kyrgyzstan is a relatively new initiative, designed to apply scientific advancements in the regions extending the most suitable conditions for the crop. An open‐field study has shown the crop's good adaptability to high‐altitude climates and specific temperature patterns on highlands in Issyk‐Kul, Talas and the foothills of Jalal‐Abad regions. Higher seed yields were achieved by the two quinoa varieties Regalona and Titicaca in highland areas, producing up to 3 tonnes of quinoa seeds.^73^

More recently, quinoa trials were conducted in different agroecological zones of Tajikistan plains, which is close to the Pamir mountainous area, indicating that the potential of expanding quinoa cultivation in the local mountain agriculture conditions is very high. A multi‐year evaluation of various quinoa accessions led to the development of a new early‐maturing, high‐yielding and nutrient‐rich local variety named ‘Vahdat’. This variety has been submitted to the State Variety Testing Committee in Tajikistan for extensive trials across diverse environmental conditions.^74^

A recent study demonstrated that quinoa can achieve competitive yields and high grain quality under the environmental conditions of Russia's Non‐Chernozem zone. Its strong adaptability and unique amino acid profile position quinoa as a crop with significant commercial potential in the region.^68^ Quinoa is recognized as a promising C3 halophyte with the capacity to enhance food security under changing climate conditions at the same time as reducing environmental stress on farmlands, yielding between 0.61 and 2.68 t ha^−1^ in high‐altitude regions when sown in spring and harvested in autumn.^69^, ^75^

The introduction and widespread adoption of suitable quinoa genotypes demands extensive efforts focused on identifying and breeding plant materials that are well‐adapted to local harsh climatic conditions.

### Breeding quinoa for marginal environments

Coffman and Smith define plant breeding for sustainable agriculture as fitting cultivars to an environment instead of altering the environment.^76^ Especially for marginal environments, where cultivars bred under optimal conditions for broad adaptation perform less well, decentralized selection in the target environment is a promising strategy to exploit specific adaptation.^77^ Decentralized selection is an option not only for marginal environments, but also for low‐input systems.^78^ Requirements for increased response to selection include the evaluation of large populations to increase selection intensity, selection environments that are representative of the target environment and increasing broad sense heritability by increasing genetic variability in the selection material or improved precision of genotype evaluation.^79^ Quinoa encompasses a broad genetic diversity and phenotypically clusters into five ecotypes, which evolved in response to different environmental conditions.^11^ Genetically, several studies found quinoa to cluster into Andean highland accessions and coastal Chilean lowland accessions.^26^ More than 16 000 accessions are conserved in genebanks, but the majority of quinoa diversity and its wild relatives are preserved by smallholder farmers and communities in the Andes, connected to traditional knowledge about its cultivation and use.^34^, ^80^, ^81^ Consequently, quinoa has a broad gene pool carrying resilience against a range of harsh environmental conditions providing the potential to find germplasm adaptable to marginal environments of different types around the world. Because quinoa is not part of the first annex of the International Treaty on Plant Genetic Resources for Food and Agriculture yet, legal frameworks for exchange of germplasm to allow screening of sufficient genetic diversity remain a challenge. However, the accessions publicly available so far provide a foundation to start quinoa breeding for marginal environments worldwide. One aspect of decentralized breeding programmes can be participatory plant breeding, which includes farmers and end‐consumers in the breeding process.^82^ Participatory breeding programmes have successfully been employed to improve local quinoa cultivars in Bolivia and inform breeders about farmers' needs in Ecuador.^83^, ^84^ In both scenarios, the programmes relied on farmers' existing knowledge about quinoa, which is not applicable to areas where quinoa has to be introduced as a novel crop. Another concept to exploit specific adaption in such cases is evolutionary participatory breeding (EPB), where populations resulting from crosses are planted repeatedly in a target environment without active selection for several generations, followed by selection on the populations by farmers, which may result in improved populations or advancing lines from them. This approach has been tested for quinoa for the US pacific north west and a consortium to promote EPB worldwide has been formed.^85^, ^86^ Given the novelty of quinoa as a crop in Central Asia, EPB managed by research institutions in collaboration with interested farmers may be an approach to simultaneously assess germplasm for local adaption and introduce farmers to quinoa production, which would help adoption of the crop upon cultivar release and integration into cropping systems.

General breeding targets in quinoa are abiotic stress tolerance, such as salt, drought, heat and cold tolerance, biotic stress tolerance, especially regarding downy mildew, and low saponin content.^87^ Besides these, yield remains the paramount criterion. Yield predictions remain difficult and are carried out based on varying indices.^88^, ^89^ Additionally, efficient phenotyping of large populations often is a bottleneck in breeding. Nevertheless, Stanschevsky *et al*.^90^ and Sosa‐Zuniga *et al*.^91^ provided extensive guidelines for phenotyping quinoa, and there has been remarkable progress in developing high‐throughput phenotyping systems for various traits in greenhouse and field settings, using a range of sensors and scales.^92^, ^93^, ^94^, ^95^, ^96^ These methods, particularly the low‐tech options, facilitate accurate phenotyping and genotype evaluation in new selection sites. Craine and Murphy^97^ have phenotyped a quinoa world core collection for agronomic and quality traits, serving as a starting point for breeders.

Quinoa is an allotetraploid species (2*n* = 4*x* = 36), resulting from the hybridization of two diploid ancestral species, *Chenopodium pallidicaule* and *Chenopodium suecium*, 3.3–3.6 million years ago.^98^ In the past 25 years, a range of molecular tools has been developed to characterize the quinoa genome,^99^ most notably the reference genome^98^, ^100^ which allows application of methods such as genome‐wide association studies (GWAS). These enable development of genetic markers to facilitate breeding for specific traits. In the same context, means of biotechnology are discussed to accelerate conventional breeding methods. Although genetic transformation of quinoa is not yet widely adopted, biotechnology holds promising opportunities for future variety development.^101^ This requires high investments and is challenging where marginal environments come with economical constraints, yet bears huge potential for sustainable agriculture.^102^

From a breeding perspective, the existing genetic diversity, breeding concepts and increasing number of phenotyping and molecular tools provide a solid foundation to enable establishing quinoa as a crop in marginal environments, if the resources available are used efficiently and effectively. However, despite initial experiences with quinoa in Central Asia, there is still a gap in translation from molecular research on adaptive traits and general environmental considerations to stable field‐level performance, which will require further agronomic research. Whether the potentials identified on an environmental level can contribute to actual sustainable agricultural production depends on whether quinoa production is adopted by farmers and whether it is also economically sustainable and socially accepted as a new crop. These aspects are discussed in the following.

## Economical aspects

### Economical farming on marginal land

Economical farming on marginal land requires a holistic approach: selecting resilient crops (e.g. quinoa), optimizing soil and water management, using appropriate fertilization and planting techniques, and ensuring institutional and market support. This integrated strategy enhances productivity and profitability despite challenging conditions. Encouraging the cultivation of pseudocereals by small and medium‐sized farmers represents an interesting strategy to promote food security in developing countries because they adapt well to different environmental conditions.^103^

Converting marginal or abandoned croplands to tree plantations can improve land productivity, enhance soil fertility and sequester carbon, thereby contributing to both local development and global climate goals, as reported in a study by Djanibekov and Khamzina^104^ based on data from Uzbekistan. Such afforestation in drylands could be a viable strategy for ecological and socio‐economic benefits, but requires integrated approaches that address both environmental and human dimensions.

The poor are often concentrated in resource‐poor areas because investments have traditionally been directed toward favoured high‐potential regions. However, these areas are increasingly prone to productivity stagnation because of the overuse of chemicals and irrigation, leading to soil erosion and salinity. At the same time, mechanization displaces labour, making investment in marginal lands both relatively more profitable and socially necessary, as argued by Fan and Chan‐Kang.^105^

The growing global bio‐based economy is expected to drive a sharp increase in biomass demand, much of which is projected to come from dedicated biomass crops. However, using fertile land for these crops raises concerns about food security. Perennial biomass crops suited for marginal lands offer a promising alternative because they can produce biomass without competing with food production. Although these crops tolerate poor conditions, their low yield and quality limit their economic viability, highlighting the need for genetic improvement‐though this is hindered by limited genetic resources. A review by Pancaldi and Trindade^106^ examines the potential of cultivating such crops on marginal lands and outlines practices to enhance their environmental and economic sustainability. They highlight approaches to plants growth and timing, efficient use of water and nutrients, and tolerance to harsh conditions. They describe the ideal plants, discuss available breeding tools and methods (including for less‐studied ‘orphan’ crops), and give examples of improvements.

Although mainly cultivated for grain, quinoa may also serve as a multipurpose crop for leaves, biomass and compounds for cosmetic industry, etc., as a byproduct, making it an interesting option to increase economic productivity on marginal lands.

Profit margins for quinoa are rather small in the main producing countries. In a study of Bolivia's quinoa sector, the survey of cooperative producers indicated that more than a half had a profit margin less than 5%, whereas 20% had only about 3% of producers.^107^

Jacobsen^19^ reports that quinoa is currently more profitable per kilogram than traditional staple crops as a result of its high market value, despite lower yields. Its profitability is especially notable in niche and health food markets, but large‐scale adoption and direct competition with other staples may be limited by supply and price factors. Jacobsen^19^ notes that quinoa commands a significantly higher market price than most traditional cereals. For example, in the USA, the grower price for quinoa is about US$1.8 kg^−1^, with retail prices for dehulled seeds around US$4 kg^−1^. In Denmark, consumer prices can reach approximately US$6 kg^−1^. These prices are substantially higher than those typically received for staple crops such as wheat or rice. Average yields for quinoa vary by region: about 1000 kg ha^−1^ in Colorado (USA), up to 3960 kg ha^−1^ in Greece, and as high as 4000 kg ha^−1^ in Kenya under favorable conditions. Although these yields are generally lower than those of major cereals (e.g. wheat or maize), the higher market price compensates for the lower yield per hectare. Quinoa is considered a ‘gourmet’ or ‘novelty item’ in developed countries, contributing to its high price and profitability. However, widespread adoption by large food companies is limited by supply constraints and the need for lower prices and reliable sourcing.

Quinoa's adaptability to marginal and drought‐prone environments could make it a more profitable option in areas where traditional cereals struggle to yield well.

Productivity and profit margins may decline over time, as observed in Bolivia, which is one of the top quinoa‐producing countries.^37^ The expansion of quinoa cultivation into areas around the salt desert, which were previously covered with natural vegetation, has led to environmental degradation. These lands are increasingly being converted into deserts because intensive farming practices deplete soil fertility and accelerate land degradation.

A study in Morocco by Hirich *et al*.^108^ examined the costs and profit margins of quinoa production, finding that quinoa is generally net‐profitable under most scenarios. The output price emerged as the most critical factor influencing profitability. The probability of financial losses was estimated to be very low, suggesting that quinoa cultivation is viable even for smallholder farmers as it can ensure a minimum income – even in dry years. Consequently, selecting and adapting genotypes with strong potential for widespread adoption across diverse agroecological zones and marginal environments is essential.

A study by Risselborn^109^ on the role of middlemen in quinoa value chains in Bolivia found that the market is highly competitive, with middlemen taking only minimal margins. As a result, quinoa producers appear to receive a fair price – one largely determined by export demand rather than by intermediaries. The study also shows that, although producers' reliance on middlemen increases with geographic distance, this does not translate into increased market power for intermediaries. Competition among middlemen remains relatively uniform across all quinoa‐producing areas.

### Adoption costs for quinoa production

The global interest in quinoa cultivation has grown significantly over the past two decades. As noted by Bazile *et al*.^14^, the number of countries cultivating quinoa increased from just eight in 1980 to nearly 95 by 2015, with the number likely to have risen further by 2025.

Introducing and expanding quinoa in new regions requires building the full value chain and training both researchers and farmers in production, harvesting, storage and processing, at the same time as fitting into existing farming systems.^15^ For example, in the Adana region in Turkey, farmers view quinoa as a crop with potential for inclusion in their cropping systems to address concerns about drought and salinity, provided that they have access to markets.^110^ For widespread adoption, strong marketing channels are essential. Because quinoa is new to most regions in the world, key elements include raising awareness, integrating quinoa into local diets and cultures, and improving its yield, nutrition and handling across the value chain.

The economic viability of quinoa cultivation is a critical factor influencing its adoption by farmers in non‐traditional growing regions. Based on experimental quinoa production trials conducted by the ICBA in Middle Eastern countries during 2019–2020,^111^ production costs and potential net profits were estimated, suggesting that a net gain is likely: in a base‐case scenario, the production cost for one hectare of quinoa with a yield of 2000 kg ha^−1^ was estimated to be around 1500 USD, with potential revenue reaching 3150 USD at the price of US$1.5 kg^−1^, resulting in a substantial net profit.

In Bolivia, one of the traditional quinoa‐producing countries, yields have remained stagnant at approximately 0.6 t ha^−1^. This contrasts sharply with Peru, comprising another major global producer, where yields have been growing and are significantly higher, reaching up to 1.6 t ha^−1^, or two to three times greater.^107^ This disparity is largely attributed to two key factors in Bolivia's quinoa production: the expansion of quinoa cultivation into marginal lands with poor soil quality, and the widespread practice of monocropping, which further degrades soil health.

In Pakistan, quinoa yields average around 3.2 tons ha^−1^. Manual production under rainfed conditions costs about US$2.80 kg^−1^, but mechanization, irrigation and organic inputs can reduce this to US$1.20 kg^−1^. There is growing interest in testing quinoa under local conditions to expand domestic markets, however, farmers face limited markets, lack of information, and insufficient technical skills.^112^

### Costs from consumption perspective

Quinoa consumption is much higher in traditional producing countries such as Bolivia and Peru, with estimated annual *per capita* consumption of 2.4 kg and 1.2 kg, respectively. By contrast, consumption in countries such as the USA has remained much lower.^113^ Even in traditional quinoa‐producing countries, economic factors affect consumption. The relatively high price of quinoa often leads people to substitute it with cheaper alternatives like pasta and rice.^114^ Although the global price spike for quinoa raised concerns about reduced access for native populations in Latin America, who may struggle to afford quinoa, evidence suggests otherwise. A study by Bellemare *et al*.,^115^ using 11 years of nationally representative household data from Peru, found that the price increase had only modest effects on the welfare of both quinoa consumers and producers.

### Social acceptability of quinoa as a new food option

Economic factors and health consciousness are key drivers influencing households to purchase and consume quinoa. Higher income levels, trust in the nutritional value of quinoa, and a greater focus on healthy eating significantly increase both the likelihood of buying quinoa and the willingness to pay for it.^114^, ^116^ Quinoa grains are highly nutritious because they contain high‐quality protein, a favourable fatty acid composition, and important vitamins, minerals and antioxidants.^117^ Additionally, it is gluten‐free and, with its high‐quality protein, it can serve as an alternative to cereals for celiac patients.^118^ For some quinoa genotypes, the amino acid profiles can meet the dietary requirements for human nutrition of all age groups.^119^ As Craine and Murphy^118^ describe, this should be considered carefully because amino acid profiles and bioavailability can change depending on the production and preparation systems. However, it points to the potential for quinoa to be part of a balanced, predominantly vegetable‐based diet.

The traditional use of quinoa in food is expanding. Although it is still commonly used in specific dishes and drinks, it is increasingly being mixed with other grains and products, particularly in the form of flour.^120^ When promoting quinoa consumption, it is important to highlight not only its nutritional value, but also its health benefits, which classify it as a functional food.^114^, ^121^ For example, rural communities in Pakistan are largely unaware of quinoa's nutritional benefits. They are unfamiliar with its taste and lack recipes to include it in traditional meals.^112^

## SOCIAL ASPECTS

### Land tenure systems in Central Asia

Introducing new crops to agricultural systems depends not only on the crop itself, but also on the existing agricultural contexts, including land tenure systems.

The success of reforms in the agricultural sector in transition countries depends on the government's commitment to provide agricultural producers with property rights such as secure land ownership.^122^ The agriculture sector plays an important role in Uzbekistan, making up around 25% of GDP.^123^ Despite ongoing land reforms in Uzbekistan, Kurbanov *et al*.^124^ argue that farmers continue to operate within a system characterized by insecure land tenure and pervasive government control. This sustained intervention, particularly in the production of cotton and wheat, restricts farmers’ decision‐making autonomy and exacerbates tenure insecurity. Given the significant links between land tenure, social well‐being and economic development in Uzbekistan, it is critical to examine the historical progression of land reform policies, which we have provided in Appendix A.

A key element, when comparing land property rights (Table 2), it can be seen that Uzbekistan is ranked the last one among the Central Asian countries. There is only land use right for farmers; however it is impossible to transfer or to own the land privately. By contrast to larger agricultural entities, such as individual farms, enterprises and clusters, which are subject to production plans and must balance state directives with discretionary land use, households (dekhan farms) possess greater flexibility in crop selection and decision‐making, facilitating the adoption of crops such as quinoa.

**Table 2:** Varieties of land property rights in Central Asian countries

Countries	Individual use right	Transfer of use right	Individual ownership right	Transfer of ownership right
Kazakhstan	+	+	−	−
Kyrgyzstan	+	+	+	−
Turkmenistan	+	−	+	−
Tajikistan	+	+	+	−
Uzbekistan	+	−	−	−

*Note:* Based on Swinnen and Rozelle (2006)^122^ and Lerman and Sedik (2018).^125^

### Migration facing land degradation

Institutional constraints and environmental degradation have limited farmers' ability to adapt, contributing to widespread outmigration from marginal areas. There appears to be considerable literature on outmigration due to environmental issues in regions in Central Asia such as Karakalpakstan. However, there is comparatively little research on the return migration to marginal lands. Some studies, either in general terms or focused on specific cases, highlight potential decisive factors that could make these marginal areas economically attractive, thereby potentially encouraging people to return.

Adaptive strategies to address climate‐induced migration exist but are often limited in the Global South due to socioeconomic constraints.^126^ In Central Asia, there is evidence of people moving from Karakalpakstan in Uzbekistan to eastern Uzbekistan and Kazakhstan as a result of environmental deterioration, with about 5–10% of the working age population being reported as leaving the region.^127^, ^128^ Since 2000, more than 500 000 people have left Karakalpakstan, consistently placing the region among the top five in Uzbekistan for out‐migration.^129^

Land degradation poses a major challenge to sustainable development and poverty reduction. A study by Mirzabaev *et al*.^130^ estimates its impact on agricultural profits in Kazakhstan, Kyrgyzstan, Tajikistan and Uzbekistan using a production function approach and unique household survey data combined with remote sensing. The results show that land degradation over the past 30 years has reduced net farming profits by 4.8 times during the 2009–2010 season. All types of households, both rich and poor, were affected. Surprisingly, poorer households adopted more sustainable land practices, driven by their reliance on land for livelihoods. This suggests poverty does not necessarily lead to degradation, although lifting poor farmers out of poverty through land management alone may require broader policy support.

Williams and Paudel^131^ investigated the impact of migration and remittance receipt on the adoption of sustainable practices (i.e. crop rotation, using combination of different crops, alternative period of cropping, etc.) in Nepal. They find that, although remittances did not directly influence adoption, migration had a significant negative effect. The study suggests that households experiencing labor shortages as a result of out‐migration should be supported with incentives to adopt effective and low‐labor conservation practices.

A study by Djanibekov^132^ examines land degradation and rehabilitation in Uzbekistan's Amu Darya River lowlands using a socio‐ecological systems framework. Cotton, a key export, has driven unsustainable land management practices, leading to widespread degradation, declining crop yields and reduced incomes. Although afforestation offers a potential solution, it is rarely implemented because of entrenched cotton‐focused policies, institutional lock‐ins and farmers’ uncertainty about the benefits of alternative land uses. These perceptions are shaped by decades of agricultural directives and investments in cotton infrastructure. The strong interdependencies among policies, institutions and production actors hinder shifts toward sustainable practices. Understanding these systemic constraints is essential for designing policies that support the adoption of innovative, sustainable land management strategies.

In the Aral Sea Basin, quinoa presents a strategic potential for climate‐resilient agriculture. As discussed earlier, it may be grown on degraded sites where traditional crops cannot because of its remarkable resistance to drought and salinity. Quinoa can be included into local farming systems to boost agricultural revenue, mitigate migratory pressures and restore agricultural output. This potential may encourage return migration by making agriculture a viable livelihood option once again.

## CONCLUSIONS

Quinoa's broad genetic diversity provides substantial potential for adaptation to hostile conditions, including these increasingly featured by marginal environments facing climate change. Considering that quinoa production can be economically viable in many scenarios, and regarding the positive impact of sustainable agricultural practices in resource‐poor environments, the integration of quinoa into existing cropping systems offers opportunities not only to increase and diversify agricultural production, but also to improve livelihoods in resource‐poor regions. In Central Asia, initial experiments indicate promising prospects for quinoa cultivation in mountainous areas, whereas harsher regions such as the Aral Sea basin remain a challenge. Although breeding tools and germplasm resources are available and continuously improved, their effective utilization remains essential. Establishing quinoa as a component of sustainable agriculture also requires the development of value chains, appropriate post‐harvest processing and market access.

Therefore, to realize the full potential of quinoa for sustainable agriculture in marginal environments, coordinated actions regarding all three components of sustainability are necessary. This means interdisciplinary strategies and institutional support spanning plant research from molecular to field level, development of infrastructure and markets and integration of social perspectives are required simultaneously. We propose these may include policy frameworks to allow crop diversification, investments in plant breeding and extension services, improvements in processing and retail infrastructure, and efforts to raise public awareness of quinoa as a nutritious food option.

## CONFLICT OF INTEREST STATEMENT

The corresponding author is at the time of submission of the manuscript a Guest Editor for the Special Issue and Editor in Chief of JSFA. No further conflict of interest is declared.

## Data Availability

Data sharing not applicable to this article as no datasets were generated or analysed during the current study.

## Appendix A

Uzbekistan has implemented multiple land reform initiatives following its independence from the Soviet Union in 1991.

Below, we summarize the periods of land reforms which cover the farm categories and policy objectives (Table A1). Every period includes the specific components of transformation based on farm types and policy objectives.

**Table A1:** Uzbekistan's land reforms since 1991

	Period	Farm types	Policy objectives
1	State farm decollectivization (1992–1997)	Kolkhozes, sovkhozes	Wheat area expansion, state farm reorganization
2	Fragmentation (1998–2002)	Shirkats, individual farms, dekhan farms	Specialization of established individual farms
3	Complete fragmentation (2003–2008)	Shirkats, individual and dekhan farms	Crop diversity and livestock farm development
4	Consolidation (2009–2015)	Individual farms and enterprises	Cotton fields relocation
5	Specialization (2016–2019)	different specialized individual farms	cotton and wheat fields relocation, diverse farms
6	Consolidation (2019‐present)	Individual farms, clusters	Cotton and wheat fields relocation

*Note:* Authors based on Djanibekov *et al*. (2012)^1^, Zorya *et al*. (2019)^2^, Babadjanov and Petrick (2023)^3^, Akhmadiyeva and Herzfeld (2021)^4^ and Kurbanov *et al*. (2025)^5^.

The initial phase of land reform in Uzbekistan focused on the reorganization of former sovkhozes into kolkhoz‐style agricultural collectives. This restructuring aimed to expand the area dedicated to wheat cultivation, with the stated objective of achieving national self‐sufficiency in grain production^6^. A subsequent period, commencing with the adoption of key legislation in 1998, witnessed the proliferation of shirkats (agricultural cooperatives), individual farms and dekhan farms (smallholder farms). This phase was characterized by a transition in land ownership, shifting from exclusive state control towards a system of collective ownership. However, Niyazmetov *et al*.^7^ note that shirkats often exhibited operational inefficiencies due to factors such as inadequate member motivation, frequent government interference in management decisions, deficient financing mechanisms, and unsustainable production pricing policies.

The third period of land reform involved the large‐scale establishment of individual farms; however, the state procurement system, mandating production quotas for cotton and wheat, persisted within the agricultural sector. Shirkats engaged in crop production were fully converted into individual farms. This period also saw increased emphasis on crop diversification, with policies designed to facilitate the development of multi‐profile individual farms. For example, agricultural enterprises traditionally focused on cotton production began to incorporate vineyards or fruit orchards into their operations. The fourth period phase of land reform was characterized by *insecure land property rights* practices. Under the guise of ‘optimization’, state agencies consolidated smaller farm units into larger agricultural holdings. For example, a given village might experience a reduction from 20–30 farms to only one or two.

Commencing in 2016, the fifth period of land reform involved a policy shift towards the fragmentation of large‐scale agricultural enterprises into smaller, specialized farms focused on specific crops, such as fruits and vegetables. This restructuring led to a reduction in the sown land area allocated to cotton and wheat, with a concomitant increase in land devoted to alternative crops. Despite these changes, the government maintained substantial control over the agricultural sector through the implementation of a state quota system, subsidized loans, a prescriptive crop allocation policy, and state subsidies for cotton and wheat production. These interventions limited farmers' autonomy in land use decisions and constrained their ability to optimize crop production efficiency.

The sixth period, beginning in 2019, witnessed a continuation of farm consolidation, particularly among cotton and wheat farms, alongside the integration of a cluster policy. This consolidation process resulted in land dispossession for some farms, leading to displacement of farmers who transitioned to wage labor on successor farms or sought off‐farm employment. From 2019 onward, cotton clusters were established extensively throughout Uzbekistan, representing the latest phase of agricultural restructuring. Petrick^8^ argues that the government's emphasis on cotton exports has consistently shaped restructuring measures, characterized by top‐down farm ‘optimization’ and cluster formation. Although the state quota system for cotton was abolished in 2020 amidst recent reform efforts, the prescriptive state crop allocation policy remained in effect. In 2021, sub‐leasing of farmland under specified conditions was permitted, reflecting a limited liberalization of agricultural production practices.

This practice has survived without any change till 2024. Regarding transfer of use rights, there has been a recent change according to the Presidential Decree No. 15, dated 18 January 2024. From 1 March 2024, it is possible for landowners to arrange sub‐renting practices. However, its application and operationalization in practice must be seen yet. Also, there is no open available information about farm categories, number, farm size, transfer rights and other indicators in the website of the statistics agency.

Appendix references

1. Djanibekov N, Van Assche K, Bobojonov I, Lamers JPA. Farm restructuring and land consolidation in Uzbekistan: new farms with old barriers. Eur‐Asia Stud 2012;64(6):1101–26.
2. Zorya S, Djanibekov N, Petrick M. Farm Restructuring in Uzbekistan: Analysis and Recommendations [Internet]. Washington, DC: World Bank Group; 2019 Feb. Available from: https://coilink.org/20.500.12592/x124jh
3. Babadjanov J, Petrick M. Uzbekistan's cotton clusters in the context of the industrial policy debate. Eurasian Geogr Econ 2025;66(3):354–83.
4. Akhmadiyeva Z, Herzfeld T. How does practice matches land laws in Central Asia? Land Use Policy 2021;109:105726.
5. Kurbanov Z, Djanibekov N, Herzfeld T. Land property rights and investment incentives in movable farm assets: evidence from post‐Soviet Central Asia. Comp Econ Stud 2025;67(2):396–425.
6. Ruziev K. Uzbekistan's development experiment: an assessment of Karimov's economic legacy. Eur‐Asia Stud 2021;73(7):1303–29.
7. Niyazmetov D, Soliev I, Theesfeld I. Ordered to volunteer? Institutional compatibility assessment of establishing agricultural cooperatives in Uzbekistan. Land Use Policy 2021;108:105538.
8. Petrick M. Post‐Soviet agricultural restructuring: a success story after all? Comp Econ Stud 2021;63(4):623–47.
